# Long-Term Regeneration and Functional Recovery of a 15 mm Critical Nerve Gap Bridged by *Tremella fuciformis* Polysaccharide-Immobilized Polylactide Conduits

**DOI:** 10.1155/2013/959261

**Published:** 2013-08-21

**Authors:** Shan-hui Hsu, Shan-Ho Chan, Chih-tsung Weng, Shu-Hui Yang, Ching-Fen Jiang

**Affiliations:** ^1^Institute of Polymer Science and Engineering, National Taiwan University, No. 1, Section 4, Roosevelt Road, Taipei 10617, Taiwan; ^2^Rehabilitation Engineering Research Center, National Taiwan University, Taipei 10617, Taiwan; ^3^Institute of Cellular and System Medicine, National Health Research Institutes, Miaoli County 35053, Taiwan; ^4^Department of Chemical Engineering, National Chung Hsing University, Taichung 40227, Taiwan; ^5^Department of Medical Imaging and Radiology, Shu-Zen Junior College of Medicine and Management, Kaohsiung 82144, Taiwan; ^6^Fengshan Tropical Horticultural Experimental Station, Council of Agriculture, Executive Yuan, Kaohsiung 83052, Taiwan; ^7^Department of Biomedical Engineering, I-Shou University, Kaohsiung 84001, Taiwan

## Abstract

Novel peripheral nerve conduits containing the negatively charged *Tremella fuciformis* polysaccharide (TF) were prepared, and their efficacy in bridging a critical nerve gap was evaluated. The conduits were made of poly(D,L-lactide) (PLA) with asymmetric microporous structure. TF was immobilized on the lumen surface of the nerve conduits after open air plasma activation. The TF-modified surface was characterized by the attenuated total reflection Fourier-transformed infrared spectroscopy and the scanning electron microscopy. TF modification was found to enhance the neurotrophic gene expression of C6 glioma cells in vitro. TF-modified PLA nerve conduits were tested for their ability to bridge a 15 mm gap of rat sciatic nerve. Nerve regeneration was monitored by the magnetic resonance imaging. Results showed that TF immobilization promoted the nerve connection in 6 weeks. The functional recovery in animals receiving TF-immobilized conduits was greater than in those receiving the bare conduits during an 8-month period. The degree of functional recovery reached ~90% after 8 months in the group of TF-immobilized conduits.

## 1. Introduction

Peripheral nerve injuries are commonly caused by trauma. The nerve would degrade in distal and renew in proximal portion. A common approach to regenerate the nerve is to bridge the defect by an autograft [1, 2], an allograft, or a biomaterial conduit. Autografts have been considered as the “clinical golden standard.” However, they have unavoidable disadvantages, such as the limited availability and donor-site morbidity. Allografts on the other hand require immunosuppression therapy which causes another problem in the long term. Therefore, a biomaterial nerve conduit that helps to bridge the nerve gap is highly desired for clinical treatment. Traditional conduits are made of nonbiodegradable silicone rubber. Recent efforts have been devoted in developing biodegradable conduits [3–5] with microporosity. For example, biodegradable poly(D,L-lactide) (PLA) conduits with asymmetric microporous structure were developed to facilitate peripheral nerve regeneration [6–9]. Nerve conduits can be loaded with biologically active components such as the Schwann cells [10] or factors analogous to neurotrophic factors [11, 12] to increase the peripheral nerve regeneration. Due to the hydrophobic nature of polymeric conduits, the method of loading for these components should be carefully designed so as not to decrease their bioactivities [13].

The bioactive herbal fruiting bodies of *Tremella fuciformis* are very popular in China as a medicinal remedy with nutritive and tonic actions for treating exhaustion. Attention has been drawn to the immunomodulating activities exhibited by their nonstarch polysaccharide components [14]. The polysaccharide fractions of *Tremella fuciformis* (TF) demonstrated pharmacological activities such as stimulation of the immune system [14, 15], antitumor [16, 17], hypoglycemic [18], hypocholesterolemic [19], anti-inflammatory [20], and antioxidant [21] effects. TF could induce human monocytes to produce interleukins and tumor necrosis factor in vitro [15, 22, 23]. In particular, TF (50–250 *μ*g/mL) was reported to significantly enhance the neurite outgrowth of nerve growth factor-(NGF-) induced PC12 h cells [24]. So far, there has been no report regarding the efficacy of TF on the regeneration of peripheral nerve.

TF is quite available and cost efficient. Moreover, the bioactivity of TF, unlike those of neurotrophic proteins, is less likely to be influenced by combination with a polymer. In this study, TF was immobilized on the PLA conduits after the surface was activated by air plasma treatment. The ability of TF-modified conduits to bridge a large (15 mm) critical gap defect in rats was evaluated by the magnetic resonance imaging (MRI) [25], electrophysiology, histology, and dynamic walking analysis following a period of 8 months. The bioactive TF-containing conduits were demonstrated to promote peripheral nerve regeneration in short as well as in long term.

## 2. Materials and Methods

### 2.1. Fabrication of the Asymmetric Microporous PLA Substrates

Fabrication of PLA substrates followed that described in the previous literature [8]. 10% solution of PLA (Mw 180 kDa, Cargill, USA) was prepared in 1,4-dioxane (Sigma, USA) and poured in glass dish. The dish was then placed in 40% ethanol (as the nonsolvent). The asymmetric porous structure was generated as a result of the immersion-precipitation phase inversion process. The precipitated substrate was washed in water and dried in air. The asymmetric permeability of the PLA substrate was determined by the diffusion of albumin in each direction (inflow and outflow with respect to the conduit).

### 2.2. Immobilization of TF onto PLA by Plasma-Assisted Grafting

**Figure 1:**
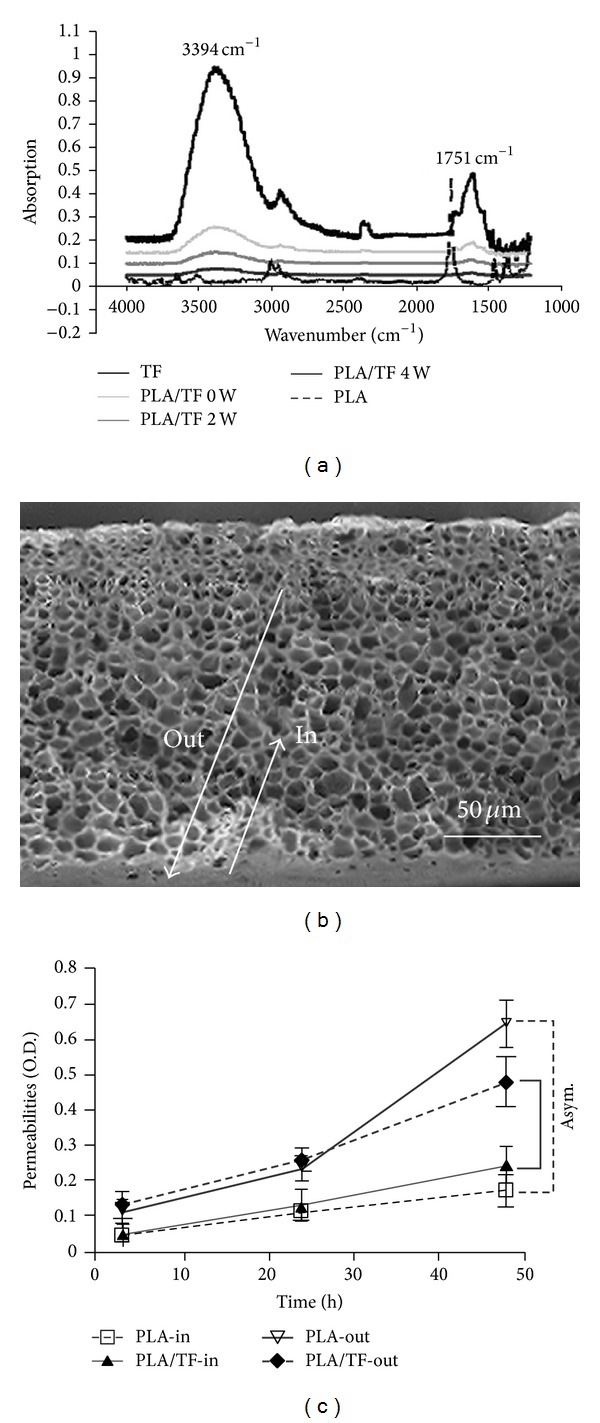
(a) ATR-IR spectra of PLA surface immobilized with TF (PLA/TF) after air plasma activation. The original spectrum is marked as “0 wk,” while spectra marked “2 wk” and “4 wk” indicate those obtained after the sample was put in PBS for 2 and 4 weeks. (b) SEM image for the cross-section of TF modified PLA. (c) The permeabilities of the original and TF-immobilized PLA substrates are shown.

TF was extracted from fruiting bodies of *Tremella fuciformis* berk above 80°C for a few hours and centrifuged at 3000–5000 rpm for about 2–5 min. The residues were resuspended in water, heated, and centrifuged repeatedly to obtain more polysaccharide extracts. Extracts obtained from this process have an average molecular weight of about 3000 kDa and are readily soluble in water due to the abundant negative charge [26]. To immobilize TF on PLA, the top surface of PLA substrate was first activated by open air plasma (Plasmatreat, Steinhagen, Germany). The parameters (power, pressure, distance, and scan rate) of plasma treatment are supplied in the Supplementary Information (see Figure 1S available online at http://dx.doi.org/10.1155/2013/959261) The TF-grafted PLA substrate prepared from the above process was abbreviated as “PLA/TF.”

The water contact angle of the modified surface was measured in the air with a static contact angle analyzer (FTA-1000 B, First Ten Angstrom Company, USA). TF grafting was confirmed by a Fourier-transformed infrared (IR) spectrometer (Perkin, Paragon-500) equipped with an attenuated total reflection unit (ATR-IR). The grafted amount was determined by gravimetry, that is, measuring the weight increase using a high sensitivity balance (Sartorius, BP211D, Germany). The surface as well as the cross-section was examined by a scanning electron microscope (SEM; ABT-150S, Topcon, USA). The samples were weighed and analyzed again by ATR-IR after placed in phosphate buffered saline (PBS) at 37°C for 2 and 4 weeks.

### 2.3. Conditions of PLA/TF by Plasma-Assisted Grafting

The plasma source was the compressed dried air (21% oxygen and 79% nitrogen) ejected from a rotating nozzle. To optimize the plasma activation process, parameters such as the plasma power, distance, and operating speed of the nozzle had to be adjusted. For the current experiment, the optimized parameters were air pressure 2.5 kg/cm^2^, plasma power 1000 W, distance 10 mm, and speed 15 m/min. Following plasma activation, the PLA substrate was immersed in 1% TF aqueous solution at 37°C for 1 h. The substrate was then rinsed with distilled water, washed extensively in an ultrasonic bath for 30 min, air-dried, and stored in a desiccator.

### 2.4. Cell Culture and Gene Expression

Rat C6 glioma cells (BCRC-60046; Bioresources Collection and Research and Research Center, Hsinchu, Taiwan) were employed for in vitro tests of the materials. C6 glioma cells were cultured in Dubeleco's modified Eagle medium (DMEM) supplemented with 44 mmol/L NaHCO_3_, 10% fetal bioactive serum, 50 U/mL streptomycin-penicillin, and 1% sodium pyruvate. Before seeding the cells, PLA and PLA/TF substrates were sterilized by 70% alcohol, rinsed with phosphate buffered saline (PBS), and placed into 24-well culture plates. C6 glioma cells at a density of 1 × 10^4^ per well were seeded. The cells were trypsinized at 24 h and 72 h, and the number was counted with a hemocytometer under an inverted microscope. A blank well (tissue culture polystyrene, TCP) was used as the control. Cells were analyzed for their neurotrophic gene expression. Total RNA was extracted from cells after 72 h. Trizol reagent (Invitrogen) was added after the cells were trypsinized. 5 *μ*g of total RNA was reverse-transcribed with the first-strand cDNA synthesis kit (Fermentas, Germany) following the manufacturer's instructions. Polymerase chain reaction was performed in a 25 *μ*L reaction volume containing 1 *μ*L of the cDNA, 0.5 *μ*L, and 10 *μ*mol/L of each primer and 5 *μ*L of 5x PCR Master Mix buffer (Gene Mark, Taiwan). Polymerase chain reaction was carried out in a GeneAmp PCR system 2700 thermal cycler (Applied Biosystems, ABI). The cycling parameters of cDNA were 35 cycles of 94°C for 30 seconds, 55°C for 30 seconds, and 72°C for 30 seconds, followed by a final extension at 72°C for 7 minutes. *β*-actin was used to confirm fidelity of the PCR reaction and as an internal control for semiquantitative analysis. The amplified products were analyzed by electrophoresis on 1.5% agarose-TAE [10 mmol/L Tris (pH 7.5), 5.7% glacial acetic acid, and 1 mmol/L EDTA] gels and visualized by ethidium bromide staining. The image was recorded by an image analyzer (BioDoc-It System, UVP, Upland, CA, USA), and the intensities of the bands were quantified by the LabWorks software. The optimized plasma parameters for PLA/TF were determined from the cell studies.

### 2.5. Animal Implantation and Evaluation

Flat substrates were rolled into conduits by the assistance of a 1.5 mm diameter mandrel. The edges were adhered tightly by a small amount of 1,4-dioxane. After rolling, the top surface of the substrates became the internal surface of the conduits. The conduits were dried under vacuum overnight to remove any residual solvent. They were checked for the dimensional fidelity and sectioned into 17 mm segments before implantation.

Forty male Sprague-Dawley rats weighing 300–350 g were used for in vivo studies. They were divided into two experimental groups. Each experimental group received PLA or PLA/TF conduits (~1.53 mm ID, ~0.21 mm in wall thickness, and ~17 mm long). The conduits were sterilized by 70% alcohol for 20 min and washed by PBS for 5 min before surgery.

Animals were deeply anesthetized with isoflurane (Halocarbon, USA) throughout the surgical procedures. Surgery was conducted on the left hind leg for each rat under aseptic conditions. After an incision had been made in the skin, the sciatic nerve was exposed by making a muscle splitting incision. A 15 mm nerve segment was excised with microscissors. The conduit (17 mm) was interposed into the 15 mm nerve defect, respectively. (The proximal nerve was anchored in the conduit by 7-0 nylon microsutures. The distal end was then sutured into the other end of the conduit. Nerve stumps at both ends were sutured into the conduit to a length of approximately 1 mm. The wound was then closed in layers using 3-0 Dexon sutures. The animals were housed in temperature-(25°C) and humidity-(45%) controlled rooms with 12 h light cycles. All procedures followed the ethical guidelines and were approved by the Animal Care and Use Committee of the university. While 15 mm gap is considered as a critical gap, results from smaller (10 mm) nerve gap (6-week) studies are included in the Supplementary Information. 

Peripheral nerve regeneration across the gap defect was monitored by the MRI technique for a long term (at 2, 4, 6, and 8 months). MRI examination was performed for 30 min. Animals were anesthetized by and maintained with isoflurane (Halocarbon, USA) throughout the imaging procedures. The MRI equipment was a 1.5 Tesla Sonata system from Simens (Germany). The image employed a 5-inch surface coil (small loop coil) to acquire the images of the transected sciatic nerve. The parameters of MRI were T1 weighted images (T1WI), T2 weighted image (T1WI), and T2 weighted short time image of inversion recovery (STIR). The time parameters were repeat time (TR) and echo time (TE). The field size was 80 mm × 80 mm. The matrix resolution was 256 × 256 pixels. All experimental parameters of MRI should be refined by different animal sizes. 

Walking track analysis was performed on all animals weekly up to 2 months and every 2 months before the animals were sacrificed. Preoperatively, the animals were trained to walk down a 150 × 8 cm track in a darkened enclosure. The sciatic functional index (SFI) that assessed the functional muscle reinnervation was calculated based on the walking track analysis, by the equation SFI = −38.3(PLF) + 109.5(TSF) + 13.3(ITF) − 8.8, where PLF (print length function) = (experimental PL − normal PL)/normal PL, TSF (toe spread function) = (experimental TS − normal TS)/normal TS (1st to 5th toes), and ITF (intermedian toe spread function) = (experimental IT − normal IT)/normal IT (2nd to 4th toes) [27, 28].

The walking behavior of rats was recorded as videos and rats were trained to walk down the 150 × 8 cm track to perform the video recordings. The walking behavior was analyzed by a semiautomation program using MATLAB 7.4.0. In the analysis, the video with the mpg/mpeg format was converted into a sequence of static images with the frames rate of frame per second. In walking behavior analysis, vector $\vec{a}$ was defined manually to select two points horizontal to the ground in the first frame. Vector $\vec{a}$ was originated from the rat ankle. Vector $\vec{a}$ had consistent directions in the rest of the frames steadily. Another vector $\vec{b}$ was defined as the central axis of the lower limb of the rat. Vector $\vec{b}$ was varied with the swing of the lower limb. The motion angle *θ* between the two vectors $\vec{a}$ and $\vec{b}$ was calculated based on the equation $\theta =\cos^{-1}(\overset{⃑}{a}\cdot \overset{⃑}{b})/(\left|\overset{⃑}{a}\right|\cdot \left|\overset{⃑}{b}\right|)$, 0° ≤ *θ* ≤ 180°. A completed swing cycle composed the changes of *θ* corresponding to the movement of the lower limb. A map indicating the different variations of *θ* versus time in terms of frame numbers could be used to evaluate the function of the limb. The extension range of the lower limb was denoted as Θ and defined as the absolute value of the gap between the maximum and the minimum of the *θ* during a swing cycle. The two parameters, Θ and the average *θ*, obtained from each map may be used to evaluate the degree of recovery of the limb after implantation of the conduit. Comparison was made to the control side (right side) of the same rat at 2, 4, 6, and 8 months.

Electrophysiological evaluation was performed before animals were sacrificed. Under anesthesia with chloral hydrate (360 mg/kg, intraperitoneal injection), the left sciatic nerve interposed by the conduit was carefully reexposed and dissected from surrounding tissues. The recording needle electrodes were placed in the anterior tibial muscle and the distal of nerve conduit. The sciatic nerve was stimulated by a pair of needle electrodes, which was placed directly on the proximal of nerve conduit and connected with DC electrical stimulator (PowerLab ML866, AD Instrument, Australia). The nerve stimulation parameter used was 1 to 10 mV and 0.2 ms duration. The ground electrode was placed in surrounding muscle tissues to remove conduction of stimulation through muscle tissues. The compound action potentials (CAPs) were recorded by a software (Scope for Windows, AD Instrument, Australia). Based on the nerve-to-nerve distances and time (from stimulation point to the maximum pulse amplitude), the nerve conduction velocity (NCV) for each group of conduits was determined.

Animals were euthanatized by CO_2_ overdose treatment after 2, 4, 6, or 8 months. The implanted grafts were harvested and immediately fixed in cold buffered 3% glutaraldehyde solution. After two days, the nerve conduits were cut open longitudinally. The specimens were then washed in PBS, postfixed in 1% osmium tetroxide (Polysciences, USA), dehydrated in a graded series of ethanol solutions, and finally embedded. The embedded samples were cut to 3 *μ*m thickness and stained with 1% toluidine blue, which did not stain PLA. All nerve sections were observed under the optical microscope, and photographs were taken using a digital camera (Nikon H666L, Japan). The cross-sectional area of regenerated nerve as well as the numbers of individual myelinated axons and blood vessels in the regenerated tissue at the midconduit was quantified by an image analysis system (Image-Pro Lite, Media Cybernetics, USA) [8]. 

### 2.6. Statistical Analysis

Forty rats underwent nerve conduit implantation. Ten rats were evaluated by MRI and sacrificed at 8 months. The average diameters of the regenerated nerve based on axial MR images at the midconduit were the mean values from the rats. The walking analysis and electrophysiological measurement were performed at 2, 4, 6, and 8 months (*n* = 5 for each group, the normal side as the control) before sacrifice for histological analysis. The results obtained from multiple samples were expressed as mean ± standard deviation. Statistical differences were analyzed by one-way analysis of variance (ANOVA). *P* < 0.05 was considered as statistically significant.

## 3. Results

### 3.1. Analysis of PLA/TF

To confirm that TF was successfully immobilized on the surface of PLA, physicochemical characterization was performed. The water contact angle of the original PLA was 76° and was reduced to 54° in PLA/TF, suggesting that the hydrophilic TF was immobilized on the surface. The corresponding ATR-FTIR spectra are shown in Figure 1(a). The original PLA surface did not show obvious absorption bands near 3394 cm^−1^ or 1751 cm^−1^. These bands were the characteristic absorption bands in TF. The surface of TF-modified PLA (PLA/TF) also demonstrated these bands, indicating that TF was successfully immobilized on PLA. Figure 1(a) also showed that the characteristic absorption bands near 3394 and 1751 cm^−1^ decreased to about one half when the TF-immobilized PLA substrate was placed in PBS for 2 weeks. These bands were small but remained visible after 4 weeks. The grafted amount of TF on PLA was 237.67 ± 40.0 *μ*g/cm^2^, based on the weight analysis. The amount of TF on the surface was 163.21 ± 32 *μ*g/cm^2^ after being placed in PBS for 2 weeks and was not detectable after 4 weeks.

The SEM images of the cross-section of the PLA/TF substrate are shown in Figure 1(b). The skin layer and the asymmetric porous structure were well kept in the modified substrate (Figure 1(b)). These changes did not influence the characteristic asymmetric permeability of the original PLA substrate (Figure 1(c)). This was important because permeability of the conduit could also affect the nerve repair.

### 3.2. Effect of TF Modification on Cell Proliferation and Gene Expression

**Figure 2:**
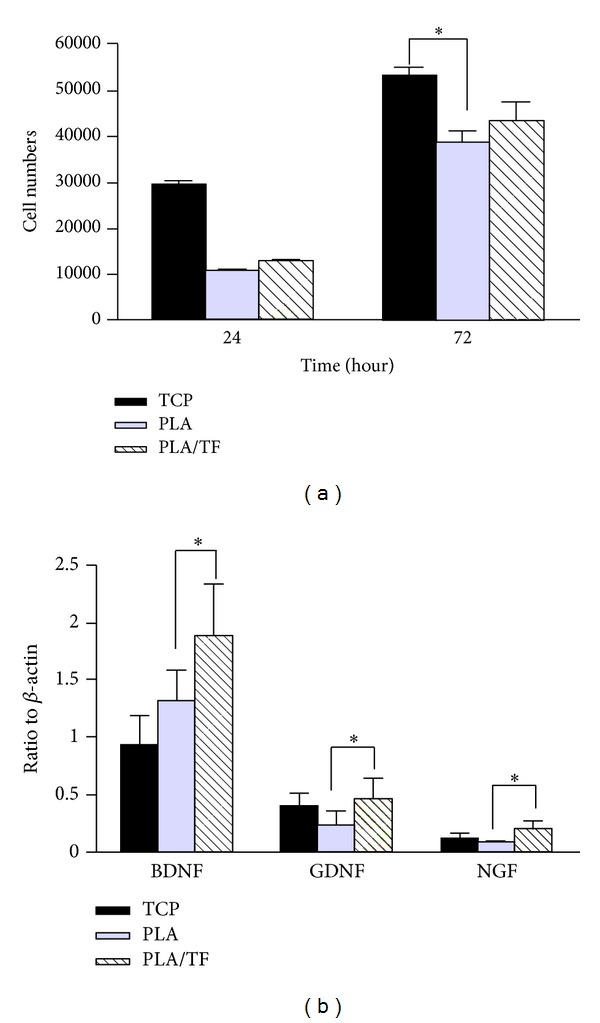
The effect of TF immobilization on the (a) proliferation and (b) gene expression of C6 cells. The gene expression was analyzed at 72 h. *P < 0.05, *n* = 3. PLA/TF: TF-immobilized PLA; TCP: tissue culture polystyrene (blank control).

The cytocompatibility was tested in vitro by analyzing the proliferation and gene expression of both C6 glioma cells on different materials. The results are shown in Figure 2. The attachment and proliferation of C6 cells on PLA and on PLA/TF were similar (Figure 2(a)). The expression of BDNF, GDNF, and NGF genes of C6 cells on PLA/TF was upregulated compared with that on PLA (Figure 2(b)). These results indicated that PLA/TF was superior to the bare PLA in promoting the neurotrophic gene expression of C6 glioma cells.

### 3.3. In Vivo Nerve Regeneration across the 15 mm Gap Defect

**Figure 3:**
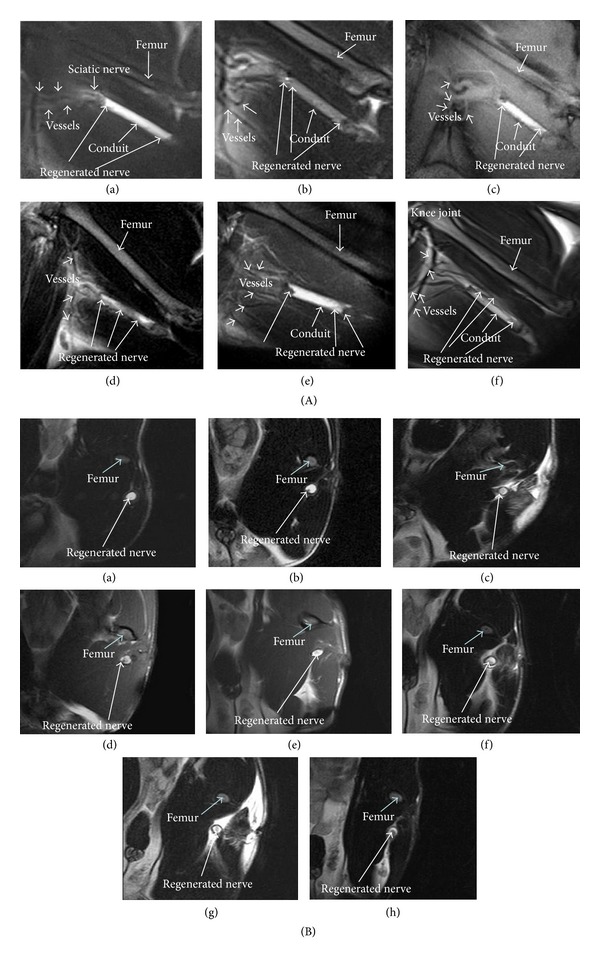
MR images for the regenerated nerve after transection (15 mm gap). (A) The sagittal views at (a), (b) 4 months, (c), (d) 6 months, and (e), (f) 8 months, for rats receiving PLA (a), (c), (e) or PLA/TF (b), (d), (f) conduits. (B) The axial views at (a), (b) 2 months, (c), (d) 4 months, (e), (f) 6 months, and (g), (h) 8 months, for rats receiving PLA (a), (c), (e), (g) or PLA/TF (b), (d), (f), (g) conduits. The regenerated nerve was indicated by long white arrows in the images. The axial views (B) were clearer than the sagittal views (A) in identifying the newly regenerated nerve.

MR images taken at different postimplantation periods are shown in Figure 3. Based on the MR sagittal views (Figure 3(A)), the sciatic nerve was at about 8 mm below the femur bone. The nerve conduit was at about 10 mm deep inside the rat body. The conduit was parallel to the femur bone. The blood vessels were observed near the distal end of the nerve conduit (indicated by the shortest arrows in Figure 3(A)). The regenerated nerves were very thin at 4 months in both PLA and PLA/TF groups (Figure 3A (a-b)). The size (diameter) of the regenerated nerve increased at 6 and 8 months (Figure 3A (c–f)). At this time, the regenerated nerve was thicker on both ends and still thin in the midsection. Based on the MR axial views (Figure 3(B)), the newly regenerated nerve inside all PLA and PLA/TF conduits was connected before 2 months (Figure 3B (a-b)). Among the five rats followed with MRI in the PLA group, the nerve of two rats was connected between 4 and 6 weeks, that is, connection not observed at 4 weeks but observed at 6 weeks. The nerve of the other three rats was connected between 6 and 8 weeks (connected nerve visualized at 8 weeks). In the PLA/TF group, the nerve in all rats was successfully connected between 4 and 6 weeks (connected nerve visualized at 6 weeks). Therefore, the success rate of nerve connection at 6 weeks was 40% for PLA conduits but was 100% for PLA/TF conduits. The newly regenerated nerves inside the conduits grew gradually thicker after connection. The average diameter of regenerative nerve in PLA/TF conduits was always greater than that in PLA conduits at each time point, as shown in Table 1.

**Figure 4:**
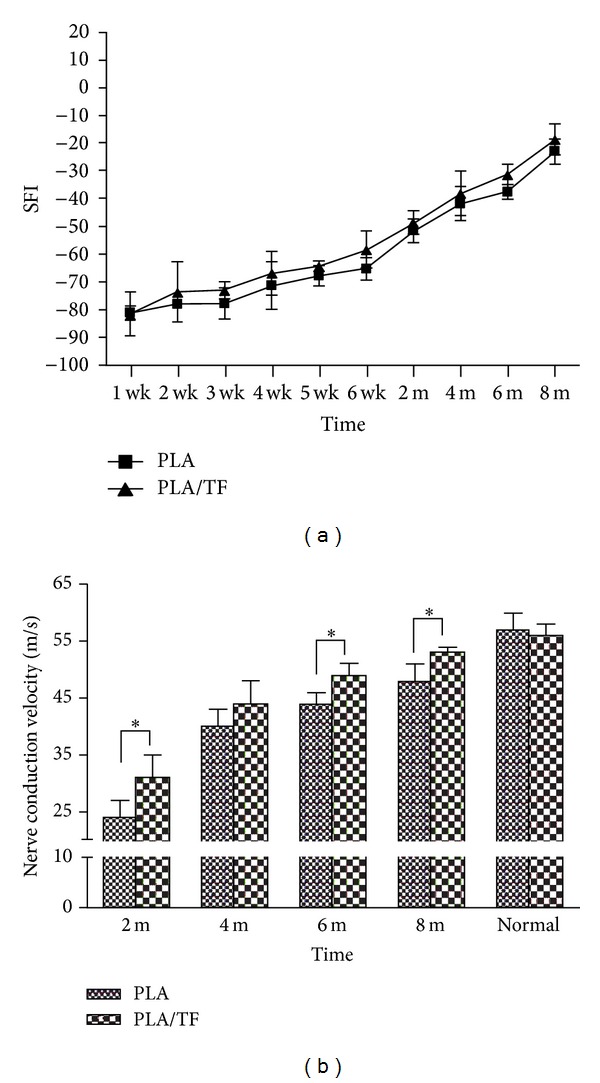
(a) SFI based on walking track analysis for rats receiving PLA and PLA/TF conduits during a period from 1 week (1 wk) to 8 months (8 m). (b) NCV obtained from electrophysiology for PLA and PLA/TF groups at different periods.

SFI data from 1 week to 8 months are shown in Figure 4(a). The value of SFI increased for both PLA and PLA/TF groups during this period. Rats receiving PLA/TF conduits showed greater SFI in average than those receiving PLA conduits during the whole period though statistical significance could not be obtained. The values of nerve conduction velocity (NCV) are shown in Figure 4(b). For the normal rat sciatic nerve, NCV was about 55 ± 3 m/s (*n* = 10). After nerve connection (2 months), the value was small for the newly regenerated nerve but increased with the time. The NCV of the regenerated nerve in the PLA/TF group was significantly larger than that in the PLA group at 2, 6, and 8 months (*n* = 5 each). The functional recovery of rats at 2 months based on the data of NCV was about 42% for the group of PLA and about 55% for the group of PLA/TF. After 8 months, the functional recovery based on NCV was 84% for the group of PLA and 93% for the group of PLA/TF.

**Figure 5:**
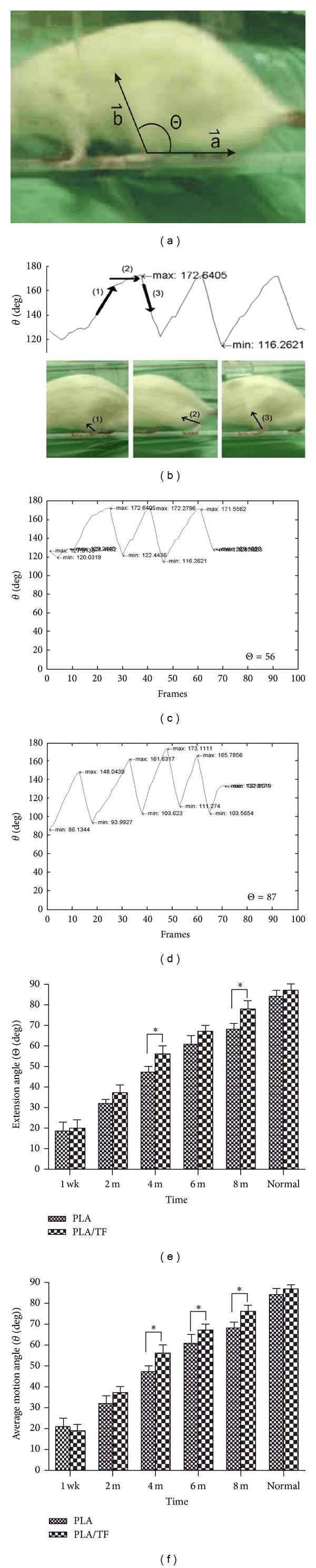
Dynamic walking analysis for rats with 15 mm transectional gap bridged by the conduit. (a) The motion angle (*θ*) was defined as the angle between vectors $\vec{a}$ and $\vec{b}$. (b) During a swing (from (1) and (2) to (3)), *θ* was recorded. Typical curves of *θ* versus frames are demonstrated for (c) the PLA/TF group at 4 months and (d) the control normal side. (e) shows the mean extension range (Θ) for PLA and PLA/TF groups at different time periods. (f) shows the mean values of average *θ* for PLA and PLA/TF groups at different time periods.

Results from dynamic walking analysis are shown in Figure 5. During a swing, the motion angle (*θ*) was recorded. The typical curves of *θ* versus frames at 4 months (for PLA/TF) and the normal limb (control side) are shown in Figures 5(c) and 5(d). The extension angle (Θ) during a swing cycle was calculated, listed with panels C-D, and summarized in Figure 5(e) (*n* = 5). The average *θ* is shown in Figure 5(f) (*n* = 5). It was obvious that the mean values of Θ and average *θ* increased from 1 week to 8 months for both PLA and PLA/TF groups. At 4, 6, and 8 months, the PLA/TF group had greater Θ as well as greater average *θ* values than the PLA group (*P* < 0.05, with one exception of Θ at 6 months where *P* = 0.07). The percent functional recovery of rats based on the restoration of Θ (versus normal) was 65% at 4 months, 77% at 6 months, and 90% at 8 months for the PLA/TF group, compared with 56% at 4 months, 72% at 6 months, and 81% at 8 months for the PLA group. These values were very close to those obtained based on the restoration of average *θ* (67% at 4 months, 80% at 6 months, and 91% at 8 months for the PLA/TF groups; 56% at 4 months, 72% at 6 months, and 81% at 8 months for the PLA group). Overall in this 15 mm critical gap rat sciatic nerve model, the PLA/TF group was superior to the PLA group in the long-term rehabilitation of walking function.

**Figure 6:**
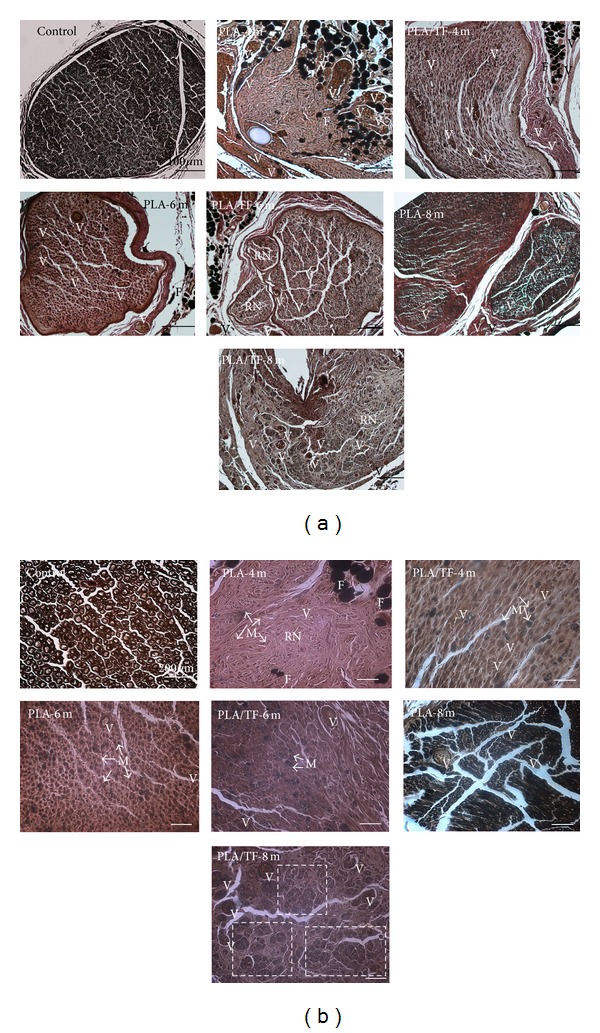
Histology for the nerve sections at the midconduit of PLA and PLA/TF conduits (15 mm gap) after 4, 6, and 8 months of implantation. (a) Images at low magnification showed the contour and shape of the regenerated tissue. (b) Images at higher magnification showed myelins and more detailed morphology. Blood vessel (V), myelin (M), fat tissue (F), and regenerated nerve (RN). Scar bars in (a) represent 100 *μ*m, and those in (b) represent 200 *μ*m. Dotted boxes in (b) indicate the formation of self-organized bundles.

Histological results of the regenerated nerve in Figure 6 showed formations of blood vessels and myelins in both PLA and PLA/TF groups at 4, 6, and 8 months. All subjects had successful connection (*n* = 5 each group). At 4 months, the myelins in the regenerated nerve were very thin. The outer contour of the regenerated nerve was irregular in shape, especially in the PLA group (Figure 6(a)). The morphology was different from the intact and homogeneous morphology of myelins in the normal sciatic nerve. The accumulation of fat tissue around the regenerated nerve was observed at 4 and 6 months but was less frequent at 8 months in both groups.

**Figure 7:**
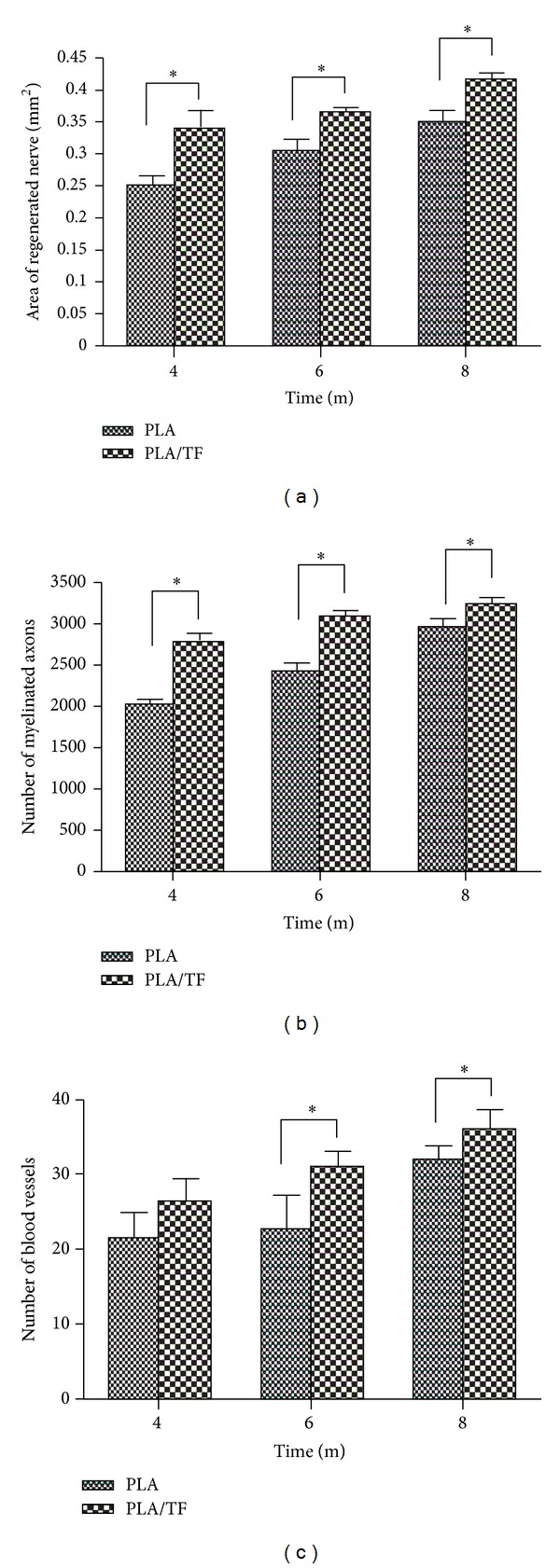
Quantitative analyses of the histological sections at the midconduit of PLA and PLA/TF conduits, showing a comparison of the (a) area of regenerated nerve, (b) number of myelinated axons, and (c) number of blood vessels between the two groups after 4, 6, and 8 months of implantation. *P < 0.05.

From 4 to 8 months, both PLA and PLA/TF groups showed improvement in the sizes of blood vessels and myelins (Figure 6(b)). The myelins were larger in size (i.e. better morphology) in the group of PLA/TF than those in the group of PLA, especially at 4 and 8 months (Figure 6(b)). Blood vessels were larger and more abundant in the group of PLA/TF, and multierythrocytes could be clearly visualized at 8 months (Figure 6(b)). At the same time, the myelins in the group of PLA/TF formed self-organized bundles, as denoted by the dotted boxes in the image. This phenomenon was not as obvious in the group of PLA. Quantitative analyses of the histology revealed a significantly larger area of regenerated nerve as well as greater numbers of myelinated axons and blood vessels in the groups of PLA/TF, as compared to PLA, during a period of 8 months (Figure 7). These results indicated that TF immobilization enhanced peripheral nerve regeneration over a critical gap defect.

## 4. Discussion

This study employed a very convenient strategy to immobilize TF on the nerve conduits by treatment with air plasma. When PLA surface was activated by air plasma, the oxygen and nitrogen in air plasma could attack the weak bonds on the surface of PLA to form free radicals. Since TF was a hydrophilic material, the water contact angle of the PLA surface decreased to 54° after TF modification. ATR-IR analysis also confirmed the grafting of TF. The current study demonstrated that TF could also be immobilized on PLA by air plasma. Besides, TF grafted on the surface of PLA was slowly released in PBS but remained to be detectable on the surface by ATR-IR after 4 weeks. This suggested that TF grafted on PLA may have endured for 4 weeks in vivo.

The rat model of sciatic nerve injury and defect was the most common animal model. The largest gap that could be created in rats was 15 mm [11]. MRI was employed in this study to monitor the long-term nerve regeneration within the conduit in the 15 mm gap nerve injury model. The images of the regenerated nerve and small blood vessels (100–200 *μ*m) in the neighborhood were obtained by the MRI technique. Evaluation of crush injury in rat sciatic nerve by MRI was recently reported in the literature [29]. In this literature, the proximal sciatic nerve of adult rats was ligated by a tight suture that was removed 1 week later to induce complete anatomy and nerve regeneration. The images in our study not only demonstrated nerve sporting from the sagittal view but also identified the cross-section of the connected nerve by the axial view. Nerve connection was directly visualized at about 2 months from the sagittal view and from the axial view. The MRI axial views also revealed that the PLA/TF group had clearer images and larger cross-section of the regenerated nerve from 2 to 8 months than those of the PLA group.

The NCV in the PLA/TF group after 4 months of implantation was over 70% of that in normal nerve. Kim et al. reported that the nerve conduction velocity of regenerated nerve through a cellular nerve graft including growth factor (nerve gap 8 mm) after implantation in rats for 6 months was about 60% [30] of that in normal nerve. The recovery degree for our PLA/TF conduits across a much larger gap (15 mm versus their 8 mm) in a shorter period (4 months versus their 6 months) was better than that previously reported.

The novel dynamic walking analysis and the resulted motion angle (*θ*) and extension range (Θ) showed the rehabilitation of walking function in rats. The data were more reliable than SFI. The SFI data had large standard deviation that statistically significant difference could not be identified between the two groups. Results from dynamic walking analysis were consistent with the tendency of SFI. Moreover, significant differences between PLA and PLA/TF groups could be demonstrated. This suggested that the analysis of extension range or average motion angle may be more appropriate to analyze the functional recovery in sciatic nerve defected animals.

Histological results showed a correlation between blood vessel formation and the number of new myelinated axons. Research has indicated that angiogenesis and neurogenesis are coupled processes [31]. TF was reported to function like NGF and promote the neurite outgrowth of PC12 h cells [24, 30]. NGF also contributes to angiogenesis [32, 33]. In our study, the vessels and myelins of regenerated nerves were evident in both PLA and PLA/TF groups at 4, 6, and 8 months. The histology of the PLA/TF group was better than that of the PLA group. While abundant blood vessels, multierythrocytes, and self-assembly of myelinated axons were usually observed in 6 weeks for the 10 mm gap [8], these features did not show up in histology until 6–8 months in the present study. It has been shown that the ligated sciatic nerve in rats recovered and had virtually the same histology as normal nerve after 12 weeks (~3 months) [11]. The evolutions and restoration of histology for the 15 mm large gap defected nerve obviously took much longer time (6–8 months in this study versus 6 weeks for the 10 mm gap). Histological results were consistent with the slow functional recovery demonstrated by the SFI, electrophysiology, and dynamic walking analysis.

In summary, our study demonstrated for the first time that TF modification was an efficient way to promote the peripheral nerve connection across a large gap in short term as well as nerve regeneration over an extensive period of time. The MRI technique successfully acquired the images of the regenerated sciatic nerve bridged by the conduits in rats. The new dynamic walking analysis provided the information of functional recovery in rats during a longer term.

## 5. Conclusion

TF was successfully grafted on the PLA nerve conduit after open air plasma treatment. The immobilization allowed TF to be retained on the conduit for more than 4 weeks. Nerve regeneration promoted by TF immobilization was evaluated in a 15 mm rat sciatic nerve transection model. MRI techniques were used to monitor the sciatic nerve regeneration within the conduit in the experimental rats during an 8-month period. TF-immobilized PLA (PLA/TF) conduits promoted the early nerve connection (6 weeks) from the two stumps. The superior efficacy of PLA/TF conduits over the bare PLA conduits was demonstrated in long term (8 months). The functional recovery after 8 months was ~84% based on electrophysiology and ~81% based on dynamic walking analysis for the bare PLA conduits, while that for PLA/TF conduits was ~93% based on electrophysiology and ~90% based on dynamic walking analysis.

## Supplementary Material

Ten male Sprague-Dawley rats weighing 300-350 g were used for the short gap (10 mm) and short term study. Five rats received conduits PLA and PLA/TF (~1.53 mm ID, ~0.21 mm in wall thickness, and ~12 mm long). After sacrifice of animals at 6 weeks, the conduits were cut open. The newly regenerated nerve was in the form of a thin white tubular substance that connected the two ends. All subjects had successful connection (n=10). Histological analysis of the regeneration nerve at the midconduit was performed and is displayed in Figure 1S. Regenerated tissue with the larger and more deeply colored myelin sheaths was observed in PLA/TF conduits. The cross-sectional area of the regenerated nerve at the midconduit of PLA/TF was more than twice larger than that of the bare PLA. The number of myelinated axons was also greater in PLA/TF conduits. By comparing the histology, it was obvious that more time (8 months vs. 6 weeks) was required for the 15-mm gap nerve defected rats to restore the same extent of morphology.

## Figures and Tables

**Table 1:** The average diameter of regenerative nerve in PLA/TF and PLA conduits determined from the axial views of MRI at 2, 4, 6, and 8 months.

Months	Nerve diameter (mm)	
	PLA/TF	PLA
2	0.4 ± 0.08	0.3 ± 0.10
4	1.0 ± 0.12*	0.7 ± 0.12
6	1.3 ± 0.16*	1.0 ± 0.08
8	2.1 ± 0.1*	1.5 ± 0.14

*P < 0.05 between PLA/TF and PLA.
