# Supplementary material for: Long-Term Regeneration and Functional Recovery of a 15 mm Critical Nerve Gap Bridged by Tremella fuciformis Polysaccharide-Immobilized Polylactide Conduits
Source: Evid Based Complement Alternat Med. 2013 Aug 21;2013:959261. doi: 10.1155/2013/959261 (PMC3763589; doi:10.1155/2013/959261)
Supplement: Supplementary file 1 — Ten male Sprague-Dawley rats weighing 300-350 g were used for the short gap (10 mm) and short term study. Five rats received conduits PLA and PLA/TF (~1.53 mm ID, ~0.21 mm in wall thickness, and ~12 mm long). After sacrifice of animals at 6 weeks, the conduits were cut open. The newly regenerated nerve was in the form of a thin white tubular substance that connected the two ends. All subjects had successful connection (n=10). Histological analysis of the regeneration nerve at the midconduit was performed and is displayed in Figure 1S. Regenerated tissue with the larger and more deeply colored myelin sheaths was observed in PLA/TF conduits. The cross-sectional area of the regenerated nerve at the midconduit of PLA/TF was more than twice larger than that of the bare PLA. The number of myelinated axons was also greater in PLA/TF conduits. By comparing the histology, it was obvious that more time (8 months vs. 6 weeks) was required for the 15-mm gap nerve defected rats to restore the same extent of morphology. [file 959261.f1.doc]

**Supplementary information**

**Short gap and short term studies**

Ten male Sprague-Dawley rats weighing 300-350 g were used for the short gap (10 mm) and short term study. Five rats received conduits PLA and PLA/TF (~1.53 mm ID, ~0.21 mm in wall thickness, and ~12 mm long). After sacrifice of animals at 6 weeks, the conduits were cut open. The newly regenerated nerve was in the form of a thin white tubular substance that connected the two ends. All subjects had successful connection (n=10). Histological analysis of the regeneration nerve at the midconduit was performed and is displayed in Figure 1S. Regenerated tissue with the larger and more deeply colored myelin sheaths was observed in PLA/TF conduits. The cross-sectional area of the regenerated nerve at the midconduit of PLA/TF was more than twice larger than that of the bare PLA. The number of myelinated axons was also greater in PLA/TF conduits. By comparing the histology, it was obvious that more time (8 months vs. 6 weeks) was required for the 15-mm gap nerve defected rats to restore the same extent of morphology.


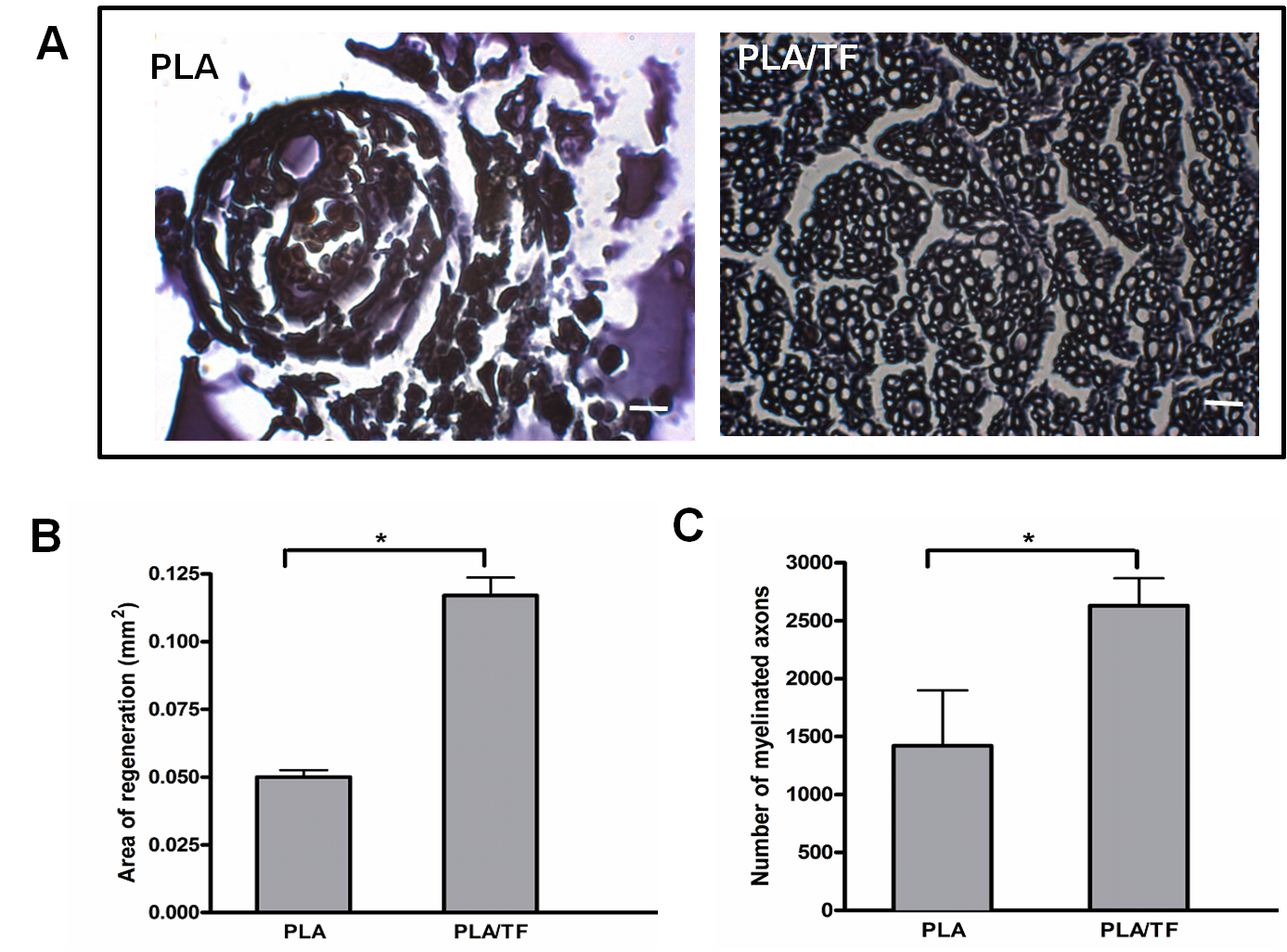


Figure 1S. (A) Histology of the regenerated nerve inside the PLA and PLA/TF conduits (at the midconduit) across a 10-mm gap at 6 weeks. Scale bar = 100 μm. (B) The area of regenerated nerve and (C) the number of myelinated axons quantified based on the histology.
